# *Oakleaf*: an *S* locus*‐*linked mutation of *Primula vulgaris* that affects leaf and flower development

**DOI:** 10.1111/nph.13370

**Published:** 2015-04-09

**Authors:** Jonathan M. Cocker, Margaret A. Webster, Jinhong Li, Jonathan Wright, Gemy Kaithakottil, David Swarbreck, Philip M. Gilmartin

**Affiliations:** ^1^ School of Biological SciencesUniversity of East AngliaNorwich Research ParkNorwichNR4 7TJUK; ^2^ John Innes CentreNorwich Research ParkNorwichNR4 7UHUK; ^3^ The Genome Analysis CentreNorwich Research ParkNorwichNR4 7UHUK

**Keywords:** heterostyly, KNOX genes, *Oakleaf*, *Primula vulgaris*, *S* locus

## Abstract

- In *Primula vulgaris* outcrossing is promoted through reciprocal herkogamy with insect‐mediated cross‐pollination between pin and thrum form flowers. Development of heteromorphic flowers is coordinated by genes at the *S* locus. To underpin construction of a genetic map facilitating isolation of these *S* locus genes, we have characterised *Oakleaf*, a novel *S* locus‐linked mutant phenotype.
- We combine phenotypic observation of flower and leaf development, with classical genetic analysis and next‐generation sequencing to address the molecular basis of *Oakleaf*.
- *Oakleaf* is a dominant mutation that affects both leaf and flower development; plants produce distinctive lobed leaves, with occasional ectopic meristems on the veins. This phenotype is reminiscent of overexpression of Class I *KNOX‐*homeodomain transcription factors. We describe the structure and expression of all eight *P. vulgaris* PvKNOX genes in both wild‐type and *Oakleaf* plants, and present comparative transcriptome analysis of leaves and flowers from *Oakleaf* and wild‐type plants.
- *Oakleaf* provides a new phenotypic marker for genetic analysis of the *Primula S* locus. We show that none of the Class I PvKNOX genes are strongly upregulated in *Oakleaf* leaves and flowers, and identify cohorts of 507 upregulated and 314 downregulated genes in the *Oakleaf* mutant.

## Introduction

Observations on different forms of *Primula* flowers date back nearly 400 yr (van Dijk, 1943; P.M. Gilmartin, unpublished). The development of two distinct floral forms, known as pin and thrum, attracted the attention of Darwin, who recognised and described their relevance and significance in his detailed studies of *P. vulgaris* and *P. veris* (Darwin, 1862). *Primula* produce either pin or thrum flowers, which exhibit reciprocal herkogamy and show different degrees of self‐incompatibility (Darwin, 1862, 1877). Pin flowers have a long style with the stigma at the corolla mouth and anthers attached midway down the corolla tube; thrum flowers have anthers which are positioned at the corolla mouth and a short style which presents the stigma midway up the corolla tube (Darwin, 1862; Webster & Gilmartin, 2006). Elevation of the anthers in thrum flowers is caused by increased cell division in the corolla tube below their point of attachment, whilst in pin flowers the style is extended by increased cell elongation (Heslop‐Harrison *et al*., 1981; Webster & Gilmartin, 2006). Differential floral architecture is orchestrated by different cellular mechanisms affecting anther elevation and style elongation (Webster & Gilmartin, 2006). Other morph‐specific differences include pollen size, corolla opening diameter, stigma shape, stigmatic papillae length and style cross‐section (Darwin, 1877; Haldane, 1933; Dowrick, 1956; Dulberger, 1975; Heslop‐Harrison *et al*., 1981; Richards, 1997; Webster & Gilmartin, 2006). Darwin observed that within‐morph pin–pin or thrum–thrum crosses were less fertile than intermorph pin–thrum or thrum–pin crosses (Darwin, 1877). This observation is underpinned by the presence of a sporophytic incompatibility system that in combination with the structural differences between the two forms of flower inhibits self‐pollination and promotes outcrossing (Shivanna *et al*., 1981; Richards, 1997).

Floral heteromorphy in *Primula* is controlled by the *S* locus; pins are homozygous recessive (*s*/*s*), thrums heterozygous (*S*/*s*) (Bateson & Gregory, 1905; Haldane, 1933; Dowrick, 1956). Studies by Ernst (Ernst, 1928, 1933) and others (Pellow, 1928; Haldane, 1933; Dowrick, 1956; Lewis & Jones, 1993) suggested that the *S* locus comprises three dominant genetic functions: *G*, which suppresses style elongation; *P*, responsible for enlarged pollen; and *A*, which controls anther elevation. These genes represent a co‐adapted linkage group. Other genes responsible for male and female sporophytic self‐incompatibility functions are also linked (Lewis, 1949; Lewis & Jones, 1993; de Nettancourt, 1997; Richards, 1997). Tight linkage of the *GPA* gene cluster maintains coupling and cosegregation of the dominant alleles. The classical model is that thrum plants have genotype *GPA*/*gpa* and pin plants *gpa*/*gpa* (Dowrick, 1956; Lewis & Jones, 1993; Richards, 1997).

Several genes linked to the *S* locus in *P. sinensis* and *P. vulgaris*, but not directly involved in floral heteromorphy, have been identified through analysis of mutants and phenotypic variation, including flower pigment genes (Gregory *et al*., 1923; De Winton & Haldane, 1933, 1935; Kurian, 1996), *Hose in Hose* (Ernst, 1942; Webster & Grant, 1990; Webster & Gilmartin, 2003; Webster, 2005) and *sepaloid* (Webster & Gilmartin, 2003; Webster, 2005; Li *et al*., 2008). Related studies on differential gene expression also identified genes that are differentially regulated in response to the *S* locus (McCubbin *et al*., 2006), and genes and polymorphisms located at, or close to, the *S* locus (Manfield *et al*., 2005; Li *et al*., 2007). However, the key *S* locus genes that orchestrate floral heteromorphy in *Primula* remain to be identified.

Heterostyly is not restricted to the Primulaceae but found in over 28 families (Ganders, 1979; Barrett & Shore, 2008) including *Primula* (Darwin, 1862), *Turnera* (Barrett, 1978) and *Fagopyrum* (Garber & Quisenberry, 1927). Progress has been made towards characterisation of the genes responsible for heterostyly in *F. esculentum* and *T. subulata* which both produce dimorphic flowers showing reciprocal herkogamy, as well as in *Linum grandiflorum* which exhibits stigma height dimorphism without anther height variation (Darwin, 1863; Lewis, 1943; Barrett, 2010). Studies in *T. subulata* based on a genetic map, chromosome deletion mutants and a BAC contig spanning the *S* locus (Woo *et al*., 1999; Labonne *et al*., 2008, 2009, 2010) enabled positional closing of the *s* haplotype (Labonne & Shore, 2011). In *F. esculentum* similar mapping approaches (Matsui *et al*., 2004; Yasui *et al*., 2004, 2008; Konishi *et al*., 2006), together with transcriptome sequencing, identified a candidate gene, *S‐ELF3*, for the short‐styled buckwheat phenotype (Yasui *et al*., 2012). Molecular analysis of protein and transcript profiles in long‐styled and short‐styled *L. grandiflorum* flowers also identified candidates for the control of dimorphic style development (Ushijima *et al*., 2012). The polyphyletic origin of heterostyly and the different floral architectures in different species suggest different molecular mechanisms underpinning heterostylous flower development. Parallel analyses of different heterostylous species are therefore important to facilitate comparative analyses of mechanisms that evolved to promote outbreeding.

A key step towards defining the key *S* locus genes in *Primula* is the identification of genetic markers for the *S* locus. Here we describe a new *S* locus‐linked mutant phenotype, which we call *Oakleaf*. We explore the molecular basis of *Oakleaf* through a candidate gene approach, and via transcriptomic and genomic analyses to profile the molecular phenotype as a prelude to construction of a genetic map of the *Primula S* locus. *Oakleaf* provides an important marker that will facilitate identification of key genes orchestrating distyly in *Primula*.

## Materials and Methods

### Plant material and linkage analysis

Plants used in this study are wild‐type *Primula vulgaris* Huds. and derived commercial cultivars. *Primula vulgaris Oakleaf* plants were originally obtained from Richards Brumpton (Woodborough Nurseries, Nottingham, UK) in 1999 and maintained by Margaret Webster as part of the National Collection of Primula, British Floral Variants. Plants were grown as described previously (Webster & Gilmartin, 2006). *Hose in Hose*,* Jack in the Green* and *Jackanapes* (Webster & Grant, 1990; Webster & Gilmartin, 2003) were crossed with *Oakleaf*, and controlled crosses between *Oakleaf* and wild‐type were performed, in insect‐free environments following emasculation of pollen recipients by removal of corolla and anthers. Seed was harvested from ripe seed capsules and stored at *c*. 4°C in air‐tight containers.

### Scanning electron microscopy (SEM)

Floral apical meristems and developing buds were dissected using scalpels and razor blades with a ×20 hand lens. Samples were prepared for cryo‐SEM, analysed and images recorded as described previously (Webster & Gilmartin, 2003).

### Draft genome sequence acquisition

Paired‐end and mate‐pair genomic DNA sequence reads were generated by Illumina HiSeq2000 at The Genome Analysis Centre, Norwich Research Park, Norwich, UK. DNA was isolated from leaves of inbred self‐fertile long homostyle *P. vulgaris* originating from Wyke Champflower, Somerset, UK (Crosby, 1940) for paired‐end read sequencing. This genotype was chosen due to homozygosity compared with outbreeding pin and thrum plants. The assembly was scaffolded with mate‐pair reads from a 9 kb thrum genomic DNA library. The paired‐end reads provided ×60 genome coverage, and the mate‐pair reads provided ×26 read coverage after filtering. A draft assembly was generated using ABySS v1.3.4 (Simpson *et al*., 2009) (kmer length = 81) to assemble paired‐end reads, then SOAPdenovo v2.0.4 (Luo *et al*., 2012) to scaffold contigs using mate‐pair reads (kmer length = 71). This process generated an assembly of 424 Mb comprising 102 442 sequences and a scaffold N50 of 47.8 kb. This draft assembly was used to identify the full complement of *PvKNOX*‐like sequences and gene model assemblies for differential transcript analysis. Full details of the fully assembled and annotated *P. vulgaris* genome will be published elsewhere.

### Gene model predictions for *P. vulgaris KNOX* (*PvKNOX*) genes

*Arabidopsis thaliana* KNOX proteins, KNAT1, KNAT2 (Lincoln *et al*., 1994), KNAT3, KNAT4, KNAT5 (Serikawa *et al*., 1996), KNAT6 (Belles‐Boix *et al*., 2006), KNAT7 (Li *et al*., 2011) and STM1 (Long *et al*., 1996), were aligned to the draft *P. vulgaris* genome with Exonerate v2.2.0 (Slater & Birney, 2005) (http://ccb.jhu.edu/software/tophat/index.shtml). *Primula vulgaris KNOX* loci were identified and gene models confirmed by transcript evidence from TopHat v2.0.8 and Cufflinks v2.1.1 (http://ccb.jhu.edu/software/tophat/index.shtml; http://cole-trapnell-lab.github.io/cufflinks/) (Trapnell *et al*., 2013) and by homology of the predicted proteins to KNOX proteins from the TAIR10 protein database (https://www.arabidopsis.org/). Parameters for protein sequence comparisons were ≥ 50% identity with ≥ 30% coverage of the KNOX query sequence. Gene models were curated manually where necessary with GenomeView (http://genomeview.org/). Sequences corresponding to *PvKNL1* were initially identified on two genomic contigs. The gene model was resolved as one locus by alignment to a Trinity (http://trinityrnaseq.github.io/) (Grabherr *et al*., 2011) assembly of the same Illumina RNA‐Seq paired‐end read data from pin and thrum flower RNA, as used for the Cufflinks analysis (Supporting Information Table S1). [Correction added after online publication 9 April 2015; in this section, URLs to TopHat and Trinity have been updated.]

### Generation of the PvKNOX phylogenetic tree

Multiple sequence alignment of *Zea mays* KNOTTED1, *A. thaliana* KNOX proteins, and predicted PvKNOX protein sequences was carried out in MEGA6 using MUSCLE (Edgar, 2004; Tamura *et al*., 2013). To obtain phylogeny support, Bayesian analyses were performed using MrBayes v3.2.2 (Ronquist *et al*., 2012) and output files visualised in FigTree v1.4.0 (http://tree.bio.ed.ac.uk/software/figtree/). The mixed amino acid substitution model was used, and the first 25% of samples were discarded as burn‐in. The consensus tree was obtained after 1000 000 generations, with the average standard deviation of split frequencies below 0.01 to ensure convergence. In addition, a acid sequence alignments of predicted protein sequences were generated with Clustal Omega (http://www.ebi.ac.uk/Tools/msa/clustalo/) (Fig. S2).

### Analysis of differential gene expression between *Oakleaf* and wild‐type plants

RNA was isolated from leaves and open flowers of *Oakleaf* and wild‐type pin plants, and from mixed stage pin and thrum flowers for RNA‐Seq using Illumina HiSeq2000 (Table S1). RNA‐Seq reads were aligned to draft *P. vulgaris* genome contigs using TopHat v2.0.8 (http://ccb.jhu.edu/software/tophat/index.shtml) (Trapnell *et al*., 2012), followed by construction and merging of the transcriptome using Cufflinks v2.1.1 (Trapnell *et al*., 2013) (http://cole-trapnell-lab.github.io/cufflinks/). [Correction added after online publication 9 April 2015; in this section, URLs to TopHat and Cufflinks have been updated.] RNA‐Seq reads from mixed stage pin and thrum flowers were used for transcriptome assembly but not subsequent expression analysis. HTSeq (Anders *et al*., 2014) was used to count raw read numbers per gene using RNA‐Seq data from *Oakleaf* and wild‐type leaf and flower samples. These read counts were normalised by estimating the effective library size with DESeq v1.16.0 (Anders & Huber, 2010) which was used to carry out differential expression analysis. Genes upregulated by a ×2 log_2_ fold‐change in both *Oakleaf* leaves and *Oakleaf* flowers were characterised by BlastX analysis (*e*‐value 1 × 10^−4^) (Camacho *et al*., 2009) to identify related sequences in the TAIR10 (https://arabidopsis.org/) and NCBI nonredundant (nr) protein databases, the latter being used as an input for Blast2GO (Conesa *et al*., 2005) All sequences have been deposited in NCBI under Bioproject number PRJNA260472. [Correction added after online publication 9 April 2015; the Bioproject number has been corrected.]

## Results

### The *Oakleaf* mutant phenotype

The *Oakleaf* phenotype was identified in 1999 amongst commercial ornamental *Primula* plants. The pedigree and cultivar of *Oakleaf* are unknown. A division of the original mutant plant was obtained by Margaret Webster and an *Oakleaf* population established which was used in this study, alongside development of *Oakleaf* in polyanthus form as a commercial variety.

**Figure 1:**
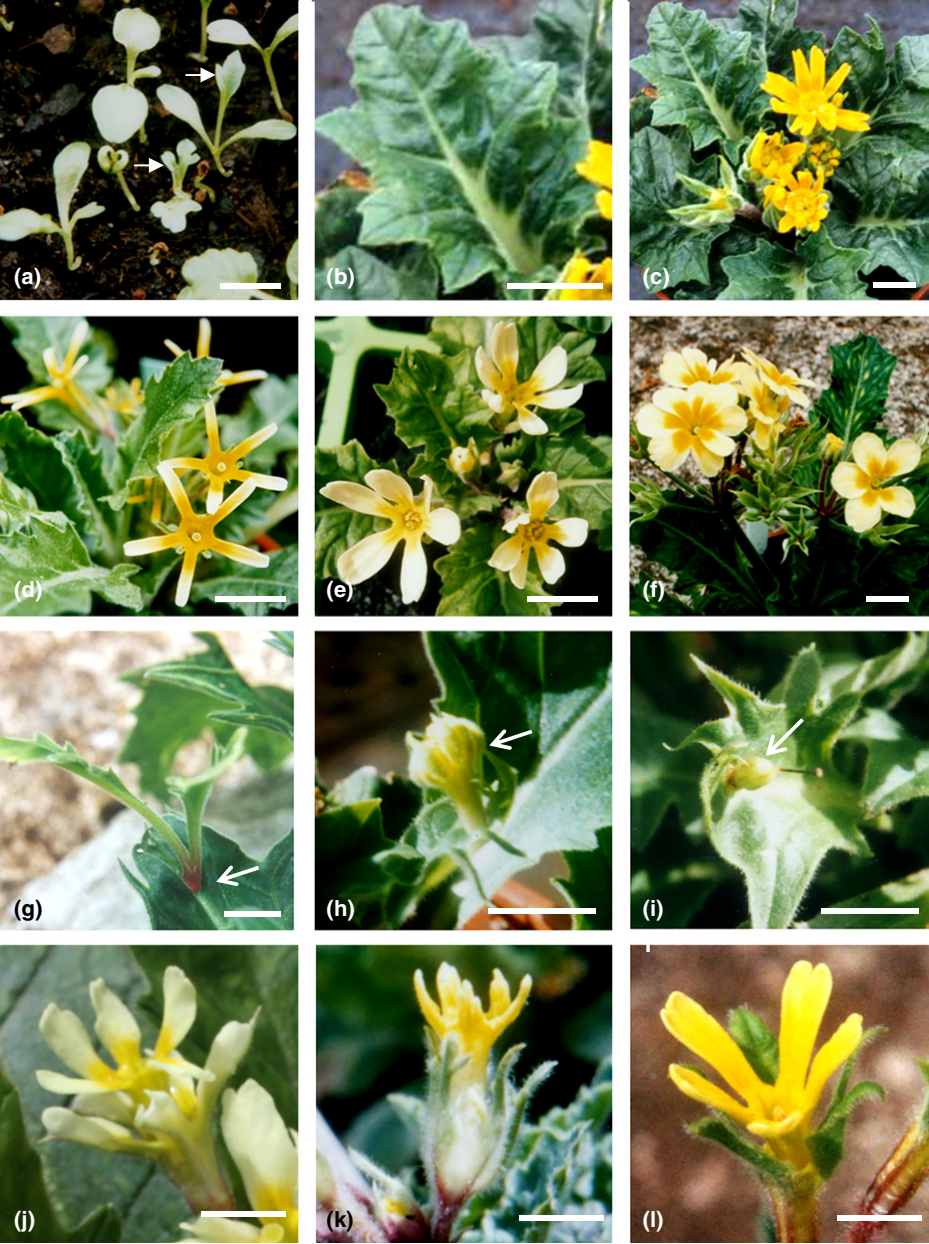
Developmental phenotypes of *Primula vulgaris Oakleaf*. (a) Seedlings from a wild‐type × *Oakleaf* cross showing wild‐type and mutant phenotypes, arrows indicate *Oakleaf* seedlings. (b) Leaf from *Oakleaf* plant. (c) *Primula vulgaris Oakleaf* mutant showing leaves and flowers. (d) Example of F_1_ plant from wild‐type × *Oak Leaf* cross showing extreme attenuated petals phenotype. (e) Example of F_1_ plant from wild‐type × *Oakleaf* cross showing partially attenuated petals. (f) Example of F_1_ plant from wild‐type × *Oakleaf* cross showing near normal petals. (g) Leaves emerging from an ectopic meristem (indicated by arrow) on the main vein of an *Oakleaf* leaf. (h) Flower bud (arrow) emerging from an ectopic meristem on the tip of an *Oakleaf* leaf. (i) Seed capsule (arrow) arising from ectopic flower shown in (h) following pollination. (j) Flower on *Hose in Hose* – *Oakleaf* double mutant plant. (k) Flower on *Jack in the Green* – *Oakleaf* double mutant plant. (l) Flower on *Hose in Hose – Jack in the Green – Oakleaf* triple mutant plant. Bars, 1 cm.

The *Oakleaf* phenotype is first visible in seedlings which sometimes produce normal and sometimes lobed cotyledons (Fig. 1a). However, the first true leaves consistently show the lobed appearance characteristic of *Quercus* species (Fig. 1a). The phenotype is variable but distinctive and easily recognisable. Mature leaves have an angular lobed appearance and contain wider and thicker leaf veins than wild‐type (Fig. 1b). The lamina of the leaf is thicker and firmer than wild‐type and the abaxial surface is pubescent. The effects of the mutation are not limited to the leaves; *Oakleaf* plants typically produce a distinctive floral phenotype. *Oakleaf* flowers are smaller than wild‐type, typically 2 cm in diameter, and calyces are frequently split (Fig. 1c) with occasional yellow petaloid material in the sepals. The severity of the floral phenotype varies as seen in the F_1_ siblings from an *Oakleaf* × wild‐type cross (Fig. 1d–f). The most extreme floral phenotype presents five narrow straight‐edged separate petals that look like the spokes of a wheel (Fig. 1d). Some plants produce an intermediate phenotype with attenuated rounded and separated petals (Fig. 1e), and in the least severe form, petals are similar to wild‐type but sometimes with splits in the corolla to give partially separated petals (Fig. 1f). *Oakleaf* plants are fully fertile as both male and female parents.

**Figure 2:**
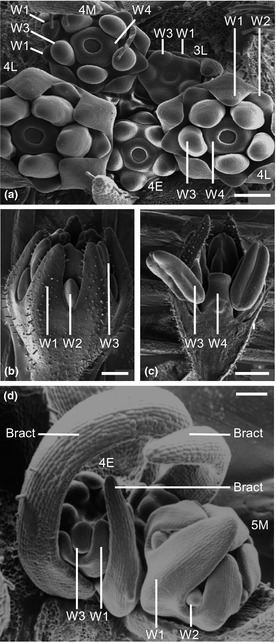
Early development of *Primula vulgaris Oakleaf* flowers. (a) Scanning electron micrograph (SEM) of *P. vulgaris Oakleaf* flowers at late stage 3 (3L), early stage 4 (4E), middle stage 4 (4M) and late stage 4 (4L). Sepal (W1), petal (W2), stamen (W3) and carpel (W4) primorida are indicated. (b) Lateral view of stage 6 *Oakleaf* flower, showing attenuation of sepals (W1) and petals (W2). Developing anthers (W3) are also visible. (c) Bisected stage 6 *Oakleaf* flower showing normal development of anthers (W3) and carpels (W4). (d) Wild‐type flowers at early stage (4E) and mid stage 5 (5M). Bracts are indicated; these were removed before cryo‐SEM from the *Oakleaf* samples shown in (a–c). Bars, 200 μm.

We previously documented wild‐type *Primula* flower development by cryo‐SEM (Webster & Gilmartin, 2003). To investigate the timing of *Oakleaf* action and any impact on early flower development, we observed *Oakleaf* flowers from late stage 3 to late stage 4 (Fig. 2a). In both wild‐type (Webster & Gilmartin, 2003) and *Oakleaf* flowers, sepals and anthers initiate at late stage 3 (Fig. 2a). Carpel development initiates at early stage 4 (Fig. 2a,d) and is accompanied by petal primordia bulges on the abaxial side of stamen primordia by mid stage 4 (Fig. 2a). *Oakleaf* does not therefore interfere with organ initiation or timing of development in early flower buds. However, at flower stage 6, the impact of *Oakleaf* on reduced petal and sepal development is visible. In *Oakleaf*, stage 6 petals are attenuated and the sepals have not expanded to engulf the developing stamens and carpels (Fig. 2b,c) as seen in wild‐type flowers at this stage (Webster & Gilmartin, 2003). Comparison to wild‐type flowers at mid stage 5 (Fig. 2d) reveals that by this earlier stage in wild‐type, the sepals have already enclosed the flower. Standardisation of developmental stage comparisons between *Oakleaf* and wild‐type were defined by equivalence of carpel development in *Oakleaf* and wild‐type flowers; *Oakleaf* does not affect carpel development. There is no difference in the *Oakleaf* phenotype between pin and thrum plants.

*Oakleaf* plants occasionally produce ectopic meristems on the veins of leaves. These ectopic meristems can be vegetative, giving rise to leaves (Fig. 1g), or floral (Fig. 1h), leading to seed pods (Fig. 1i) but without viable seeds. Some aspects of the *Oakleaf* phenotype are reminiscent of the effects of ectopic expression of Class I *KNOX* homeodomain genes in *Arabidopsis* (Lincoln *et al*., 1994; Chuck *et al*., 1996; Hay & Tsiantis, 2010), and their role during normal development of lobed leaves in tomato and *Cardamine hirsuta* (Hareven *et al*., 1996; Bharathan *et al*., 2002; Hay & Tsiantis, 2006; Shani *et al*., 2009).

In order to explore the influence of *Oakleaf* on leaf and petal development and to examine whether the effects are organ‐specific or whorl‐specific, we combined *Oakleaf* with the following mutant phenotypes: *Hose in Hose* (Webster & Grant, 1990; Li *et al*., 2010), a dominant mutant phenotype in which sepals are converted to petals; *Jack in the Green* (Webster & Gilmartin, 2003), a dominant mutant phenotype in which sepals undergo a homeotic transformation to leaves; and *Jackanapes* (Webster & Gilmartin, 2003), a double mutant carrying both *Jack in the Green* and *Hose in Hose* dominant alleles, which produces hybrid petal/leaf structures in the first floral whorl.

Progeny from crosses of *Oakleaf* and *Hose in Hose* produce flowers with two whorls of *Oakleaf* type petals (Fig. 1j); progeny from crosses between *Oakleaf* and *Jack in the Green* produce flowers with characteristic *Oakleaf* petals surrounded by a calyx of miniature *Oakleaf* leaves (Fig. 1k); progeny from crosses between *Oakleaf* and *Jackanapes* produce flowers with a corolla of *Oakleaf* petals surrounded by a calyx comprising hybrid *Oakleaf* leaves and yellow petaloid tissue (Fig. 1l). Appearance of the *Oakleaf* phenotype in combination with other mutant phenotypes in F_1_ progeny indicates that the *Oakleaf* allele is dominant to wild‐type and that its effect on petal and leaf development is determined by organ identity and not organ position.

### Inheritance of *Oakleaf*

**Figure 3:**
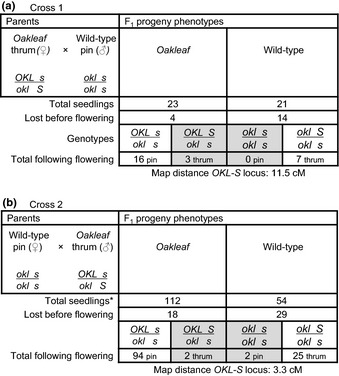
Genetic analysis of reciprocal crosses between *Primula vulgaris Oakleaf* and wild‐type plants. The results of reciprocal crosses between a *P. vulgaris Oakleaf* thrum and a wild‐type pin plant are shown. (a) Cross 1, *Oakleaf* as female parent. (b) Cross 2, *Oakleaf* as male parent. The phenotypes and genotypes, with respect to leaf shape (wild‐type or *Oakleaf*), and the *S* locus (pin or thrum) of parent plants are indicated. The phenotypes, and predicted genotypes, of F_1_ progeny are shown, along with numbers of progeny classified initially only with respect to leaf shape. The number of each class of progeny lost before flowering is shown, as well as the number of pin‐ and thrum‐type flowers found on *Oakleaf* and wild‐type plants. *Oakleaf* (*OKL*) is shown in coupling to the recessive *s* allele of the *S* locus in the original plant based on the assumption that minor progeny classes represent recombinants; genotypes of recombinant chromosomes in progeny and numbers of recombinant progeny are shaded grey. *Does not include 45 seedlings that died before forming secondary leaves.

Preliminary analyses in horticultural crosses suggested that *Oakleaf* was dominant to wild‐type (R. Brumpton, pers. comm.). Our crosses between *Oakleaf* and floral mutants reinforce this observation. To fully explore the inheritance of *Oakleaf* we undertook a series of controlled crosses. The first crosses (Fig. 3) used an *Oakleaf* thrum as both pollen recipient (Cross 1) and pollen donor (Cross 2) with a wild‐type *P. vulgaris* pin plant. Seed from the *Oakleaf* thrum parent (Cross 1) yielded 44 progeny: 23 *Oakleaf* and 21 wild‐type based on seedling phenotype (Fig. 3a); chi‐squared analysis supports a 1 : 1 ratio (*P *>* *0.70). Four *Oakleaf* and 14 wild‐type plants were subsequently lost between seedling stage and flowering. The excess of pin *Oakleaf* and thrum wild‐type progeny indicate linkage of *Oakleaf* to the *S* locus with coupling to the recessive *s* allele; three thrum *Oakleaf* progeny reveal recombination of *Oakleaf* from the recessive *s* allele to the dominant *S* allele. These small numbers suggest a map distance for *Oakleaf* to *S* of 11.5 cM.

The reciprocal cross (Cross 2) with *Oakleaf* as pollen donor, confirmed linkage of *Oakleaf* to *S and* coupling to the recessive *s* allele (Fig. 3b). Of the 258 seeds planted, 45 germinated but died before producing secondary leaves and could not be scored. Of the remaining 112 *Oakleaf* and 54 wild‐type plants, a further 18 *Oakleaf* and 29 wild‐type plants died before flowering. These data (Fig. 3b) suggest a significant deviation from the anticipated 1 : 1 ratio (*P *<* *0.001) of *Oakleaf* to wild‐type. Linkage of *Oakleaf* to the *S* locus is supported by the excess of *Oakleaf* pin and wild‐type thrum plants. Four progeny, two *Oakleaf* thrums and two wild‐type pins (Fig. 3b), are recombinants; these larger progeny numbers give a map distance between *Oakleaf* and the *S* locus of 3.3 cM.

**Figure 4:**
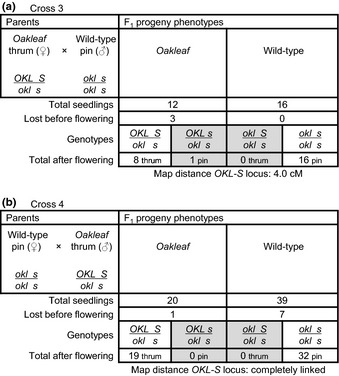
Confirmation of linkage between *Primula vulgaris Oakleaf* and the *S* locus. A recombinant *P. vulgaris Oakleaf* thrum plant was used in a reciprocal back cross with a wild‐type pin plant. (a) Cross 3, *Oakleaf* as female parent. (b) Cross 4, *Oakleaf* as male parent. The phenotypes and genotypes, with respect to leaf shape (wild‐type or *Oakleaf*), and the *S* locus (pin or thrum) of parent plants are indicated. The phenotypes, and predicted genotypes, of F_1_ progeny are shown along with numbers of progeny classified initially only with respect to leaf shape. The number of each class of progeny lost before flowering is shown, as well as the number of pin‐ and thrum‐type flowers found on *Oakleaf* and wild‐type plants. Based on data from Fig. 4, the *Oakleaf* parent used in this cross carries the *OKL* locus in coupling to the dominant *S* allele of the *S* locus; genotypes of recombinant chromosomes in progeny and numbers of recombinant progeny are shaded grey. The map distance in cM between *OKL* and the *S* locus are indicated.

In order to further confirm linkage to the *S* locus we backcrossed an *Oakleaf* thrum progeny plant to a wild‐type pin plant. With the *Oakleaf* thrum as pollen acceptor (Cross 3), 28 progeny were obtained: 12 *Oakleaf* and 16 wild‐type yielding the anticipated 1 : 1 ratio (*P *>* *0.3) (Fig. 4a). Three *Oakleaf* plants died before flowering and one *Oakleaf* plant produced pin flowers, revealing recombination between *S* and *Oakleaf* bringing *Oakleaf* back in coupling with *s* (Fig. 4a). The reciprocal cross (Cross 4) yielded 20 *Oakleaf* and 39 wild‐type plants; eight plants were lost before flowering and no recombinants were found in the remainder (Fig. 4b). These data indicate distortion of the anticipated 1 : 1 ratio (*P *>* *0.01) of *Oakleaf* to wild‐type, with *Oakleaf* progeny underrepresented. In this cross we did not observe any losses of plants at the seedling stage.

**Figure 5:**
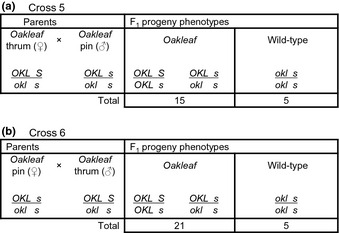
Reciprocal crosses between *Primula vulgaris Oakleaf* pin and *Oakleaf* thrum plants to assess viability of *Oakleaf* homozygotes. The results of reciprocal crosses between a *P. vulgaris Oakleaf* thrum, with *OKL* in coupling with the dominant *S* allele, and an *Oakleaf* pin, with *OKL* in coupling to the recessive *s* allele, are presented. (a) Cross 5, *Oakleaf* thrum as female parent. (b) Cross 6, *Oakleaf* pin as female parent. The phenotypes and genotypes, with respect to leaf shape (wild‐type or *Oakleaf*), and the *S* locus (pin or thrum) of parent plants are indicated. The number of F_1_ progeny of each phenotype, classified with respect to leaf phenotype, and their predicted genotypes are shown; progeny were not scored with respect to flower morph.

In order to investigate deviation from the anticipated 1 : 1 ratio of *Oakleaf* to wild‐type plants in progeny from Cross 2 and Cross 4, we undertook further analyses. Reciprocal crosses between an *Oakleaf* pin and an *Oakleaf* thrum, with *Oakleaf* in coupling to *S*, were established with the thrum as pollen donor (Cross 5) and pollen recipient (Cross 6) (Fig. 5). These crosses were predicted to yield a 3 : 1 ratio of *Oakleaf* to wild‐type plants which would be characteristic of a cross between two heterozygotes each carrying a dominant allele. From Cross 5 we obtained 15 *Oakleaf* and five wild‐type plants and from Cross 6 we obtained 21 *Oakleaf* and five wild‐type plants, both results being consistent with the expected 3 : 1 ratio (*P *>* *0.95 and *P *>* *0.50, respectively) (Fig. 5a,b).

### Characterisation of the *PvKNOX* gene family

Aspects of the *Oakleaf* phenotype – namely lobed leaves, ectopic meristems and dominance – are reminiscent of the consequences of ectopic overexpression of Class I *KNOX* genes in *A. thaliana* (Lincoln *et al*., 1994; Chuck *et al*., 1996; Hay & Tsiantis, 2010). We therefore set out to explore whether *Oakleaf* results from a constitutive overexpression mutation of a *KNOX* homeodomain gene. We considered and explored three possibilities: that the phenotype is caused by upregulation of a *PvKNOX* gene in mature leaves and flowers of *Oakleaf* plants; that the phenotype arises from a mutation in a *PvKNOX* gene that does not affect expression but confers a dominant gain of function in protein activity; that the dominant mutation is caused by upregulation of a gene unrelated to the *PvKNOX* gene family.

The *KNOX* homeodomain gene family in Maize (Vollbrecht *et al*., 1991), *Arabidopsis* (Lincoln *et al*., 1994; Long *et al*., 1996; Serikawa *et al*., 1996; Belles‐Boix *et al*., 2006; Li *et al*., 2011) and other species (Bharathan *et al*., 1999; Hay & Tsiantis, 2010) have been characterised and classified as Class I or Class II based on phylogenetic relationships and expression dynamics (Kerstetter *et al*., 1994; Bharathan *et al*., 1999). We used this framework to define the full complement of Class I and Class II *PvKNOX* genes. Illumina RNA‐Seq analysis of wild‐type *P. vulgaris* leaf and flower transcriptomes, together with transcriptome analysis of *Oakleaf* mutant leaves and flowers, was used to generate a transcriptome dataset. We also included RNA‐Seq datasets obtained from pin and thrum mixed stage flower samples to maximise the opportunity for *PvKNOX* related gene identification; these mixed pin and thrum flower RNA‐Seq samples were not included in subsequent comparative expression analyses. A summary of read number, base coverage and transcript assemblies from these six RNA samples is presented in Table S1.

**Figure 6:**
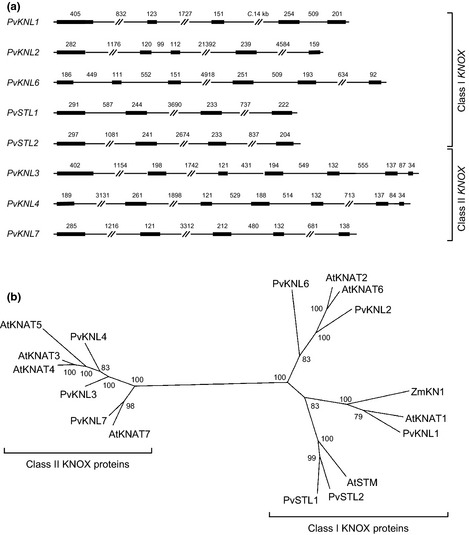
Classification of the *PvKNOX* gene family. (a) Predicted gene structures of eight *Primula vulgaris* gene models that encode proteins with amino acid similarity to the *Arabidopsis thaliana knotted‐homeodomain* (*KNOX*) gene family members *KNAT* and *STM*;* Primula* genes were named *Knotted‐Like* (*PvKNL*) and *Shootmeristem‐Like* (*PvSTL*). Gene structures are represented by thick lines (exons) and thin lines (introns); the number of bases in each intron and exon is shown. Genes are shown grouped as Class I and Class II *KNOX* genes. Accession numbers: *PvKNL1*, KM586811; *PvKNL2*, KM586816; *PvKNL3*, KM586814; *PvKNL4*, KM586817; *PvKNL6*, KM586812; *PvKNL7*, KM586810; *PvSTL1*, KM586815; *PvSTL2*, KM586813. (b) Unrooted phylogenetic tree based on amino acid sequence of the eight *P. vulgaris* PvKNL and PvSTL homeodomain proteins in comparison to *A. thaliana* KNAT and STM proteins, with *Zea Mays* KNOTTED1. Class I and Class II KNOX protein clades are identified. Posterior probabilities for clades are shown as percentages.

In parallel, we used Illumina sequencing to generate a draft *P. vulgaris* genome sequence. The full assembly and annotation of the genome will form the basis of a subsequent publication. We screened this draft genome assembly with *A. thaliana* KNOX protein sequences using Exonerate c2.2.0 (Slater & Birney, 2005) and identified nine genomic contig assemblies with *KNOX* gene homology. Within these contigs we defined gene models using the RNA‐Seq dataset with Tophat v2.0.8 (Trapnell *et al*., 2012) and Cufflinks v2.1.1 (Trapnell *et al*., 2013). Seven of the genomic contigs were predicted to contain full‐length *PvKNOX* gene models. Of the two remaining contigs, one contained three exons representing the 5′‐end of a *PvKNOX* gene, the other contained two exons corresponding to the 3′‐homeodomain region. It was not initially clear whether these models represented two partial loci or one locus split between two contigs due to an incomplete genome assembly. Both partial models were supported by RNA‐Seq data. We therefore screened a *de novo* Trinity (Grabherr *et al*., 2011) transcript assembly generated from RNA‐Seq data of the *P. vulgaris* pin and thrum mixed stage flower bud RNA samples, and identified a single Trinity transcript assembly derived from a single locus (*PvKNKL1*) bridging the unjoined genomic contigs. This finding resolved that the *P. vulgaris* genome encodes eight *PvKNOX* genes; the predicted gene structures are shown in Fig. 6(a). Figure S1 presents the predicted amino acid sequence from each gene; a Clustal Omega sequence alignment of the eight proteins with conserved protein domains indicated is shown in Fig. S2.

Figure 6(b) shows a phylogenetic analysis of the eight predicted PvKNOX proteins (Fig. S1) in comparison to the *A. thaliana* KNOX protein family, comprising seven KNAT proteins (Lincoln *et al*., 1994; Serikawa *et al*., 1996; Belles‐Boix *et al*., 2006; Li *et al*., 2011) and STM1 (Long *et al*., 1996)*,* together with KNOTTED‐1 from *Zea mays* (Vollbrecht *et al*., 1991). Following this analysis we named the *PvKNOX* genes and their encoded proteins *Knotted‐like* (*PvKNL*) and *Shootmeristem‐like* (*PvSTL*) based on encoded protein sequence similarity. *Primula vulgaris* does not have a homologue of *AtKNAT5*, but contains two *STM‐*like genes; it therefore has five Class I and three Class II *PvKNOX* genes.

### Expression analysis and sequence comparison of *PvKNOX* genes in wild‐type and *Oakleaf*

**Figure 7:**
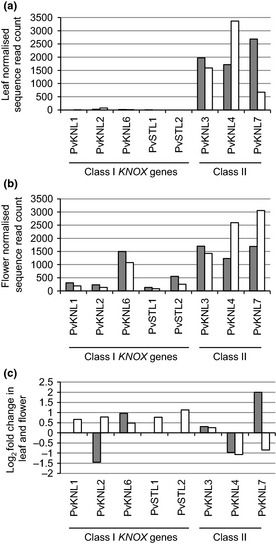
Differential expression of the *PvKNOX* gene family in *Primula vulgaris Oakleaf* and wild‐type plants. Expression of the eight genes represented by normalized Illumina RNA‐Seq read count from (a) RNA isolated from *P. vulgaris Oakleaf* leaves (closed bars) and wild‐type leaves (open bars); (b) RNA isolated from *Oakleaf* flowers (closed bars) and wild‐type flowers (open bars). (c) the Log_2_ fold increase or decrease in expression levels between *Oakleaf* leaf and wild‐type leaves (closed bars) and *Oakleaf* and wild‐type flowers (open bars). The wild‐type was a pin plant. Class I and Class II *PvKNOX* genes are indicated.

Identification of the full complement of Class I and Class II *PvKNOX* genes enabled us to compare the expression of each gene in leaves and flowers of wild‐type and *Oakleaf* to determine whether constitutive upregulation of a *PvKNOX* gene was associated with the *Oakleaf* phenotype. Based on previous studies of overexpression of Class I *KNOX* genes in other species (Smith *et al*., 1992; Lincoln *et al*., 1994; Chuck *et al*., 1996; Hareven *et al*., 1996; Bharathan *et al*., 2002; Hay & Tsiantis, 2006; Shani *et al*., 2009) we explored whether the *Oakleaf* phenotype also resulted from constitutive upregulation of a *PvKNOX*‐like gene. Our gene expression analyses, described earlier, used HTSeq to create a data file of RNA‐Seq reads aligned to each locus. We then used DESeq to compare RNA‐Seq read counts for each locus in *Oakleaf* leaves, *Oakleaf* flowers, wild‐type leaves and wild‐type flowers (Anders & Huber, 2010). Graphical representation of the data is shown in Fig. 7. Normalised read counts for each gene in each tissue, and the log_2_ fold‐change between *Oakleaf* and wild‐type leaves, and *Oakleaf* and wild‐type flowers, are shown in Table S2.

The five Class I *PvKNOX* genes are expressed at very low levels in leaves of both wild‐type and *Oakleaf* plants (Fig. 7a). Only *PvKNL2* and *PvKNL6* produce measurable read counts from leaves (Table S2). Higher expression levels are observed for the Class II *PvKNOX* genes in wild‐type and *Oakleaf* flowers (Fig. 7b). When relative expression levels are compared between *Oakleaf* and wild‐type, all Class I *PvKNOX* genes show higher expression levels in *Oakleaf* flowers than wild‐type; only *PvKNL6* shows higher read counts in *Oakleaf* leaves (Fig. 7c; Table S2), but the normalised read counts of only 15 and 7 reads, respectively, are only just above background. None of the Class I *PvKNOX* genes are strongly upregulated in *Oakleaf* leaves (Fig. 7c; Table S2).

In contrast to the Class I *PvKNOX* genes, the three Class II *PvKNOX* genes – *PvKNL3*,* PvKNL4* and *PvKNL7 –* show strong expression in both leaves and flowers of *Oakleaf* and wild‐type plants (Fig. 7a,b). Only one gene, *PvKNL3*, is upregulated in both leaves and flowers of *Oakleaf* (Fig. 7; Table S2). *PvKNL3* expression in *Oakleaf* and wild‐type leaves is represented by normalised read counts of 1971 and 1591 reads, respectively. Normalised read counts for *Oakleaf* and wild‐type flowers are 1704 and 1424, respectively (Table S2). These values give Log_2_ fold upregulation in *Oakleaf* of 0.31 for leaves and 0.26 in flower (Fig. 7c; Table S2).

It is possible that a dominant phenotype could arise through a splicing mutation that results in a protein lacking a critical regulatory domain. We therefore compared RNA‐Seq read abundance profiles across all predicted exons of all *PvKNOX* loci and saw no difference between *Oakleaf* and wild‐type that would indicate alternate splicing profiles. We did, however, identify 18 polymorphisms between seven *PvKNOX* genes in *Oakleaf* and the wild‐type *PvKNOX* sequences from the genome assembly that would cause amino acid substitutions (Table S3). The *Oakleaf* plant used was heterozygous for the *Oakleaf* locus in a pin genetic background. We therefore then compared the *Oakleaf* single nucleotide polymorphisms (SNPs) with *PvKNOX* genes expressed in the flowers and leaves of a wild‐type pin plant to determine whether the SNP was *Oakleaf*‐specific. Three SNPs in *PVKNL2* and *PvSTL1* were predicted to result in truncated proteins (Table S3). For the seven remaining SNPs in *PvSTL1*,* PvKNL3, PvKNL4* and *PvKNL7*, the potential impact of amino acid substitution was analysed using the SIFT prediction tool (Ng & Henikoff, 2003). Five SNPs were predicted to result in tolerated amino acid substitutions which would represent conservative changes (Table S3) and the remaining two, Leu^335^‐Ser in *PvKNL3* and Gly^6^‐Glu in *PvKNL7,* are predicted to result in nontolerated amino acid substitutions (Table S3) and could therefore affect protein function (Ng & Henikoff, 2003).

### Differential gene expression between *Oakleaf* and wild‐type plants

KNOX proteins are transcriptional regulators and we would therefore anticipate wider changes in patterns of gene expression of both direct and indirect target genes in response to any aberrant expression of a *PvKNOX* gene in *Oakleaf*. It is also possible that *Oakleaf* is caused by mutation of an unrelated gene that results in a similar phenotype to that predicted from overexpression of a *PvKNOX* gene. Either possibility would result in transcript profile changes between *Oakleaf* and wild‐type plants. We therefore used *Oakleaf* and wild‐type flower and leaf RNA‐Seq data to explore global transcriptome changes between *Oakleaf* and wild‐type plants.

**Figure 8:**
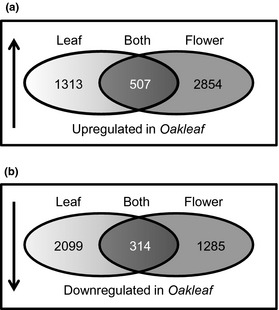
Identification of up‐ and downregulated genes in *Primula vulgaris Oakleaf* compared with wild‐type. Venn diagrams showing: (a) numbers of genes upregulated in *Oakleaf* leaves (light grey) and *Oakleaf* flowers (mid‐grey) compared with pin wild‐type leaves and flowers. The numbers of genes upregulated in both organs (dark grey) are shown. (b) Numbers of genes downregulated in *Oakleaf* leaves (light grey) and *Oakleaf* flowers (mid grey) compared with pin wild‐type leaves and flowers. The numbers of genes downregulated in both organs (dark grey) are shown.

Assembly of the RNA‐Seq datasets through alignment to the draft *P. vulgaris* genome identified a total of 39 193 transcript models and created a data file of all RNA‐Seq reads aligned to each of the corresponding loci. HTSeq and DESeq (Anders & Huber, 2010; Anders *et al*., 2014) were then used to generate normalised counts of RNA‐Seq reads corresponding to each locus for each of the four RNA‐Seq samples from leaves and flowers of wild‐type and *Oakleaf* plants. Analysis using a log_2_ fold‐change threshold > 2 identified 1313 genes upregulated in *Oakleaf* leaves and 2854 genes upregulated in *Oakleaf* flowers. Of these genes, 507 were common to both tissues. Parallel analyses using the same threshold identified 2099 genes downregulated in *Oakleaf* leaves and 1285 downregulated in *Oakleaf* flowers, of which 314 were represented in both tissues. These data are summarised in Fig. 8. None of the *P. vulgaris KNOX* genes are included in these samples as the fold‐change in expression for these genes is below the two‐fold cut‐off used. Summaries of genes which are upregulated, or downregulated, in both leaves and flowers of *Oakleaf*, including BlastX analysis of nonredundant protein and Arabidopsis TAIR databases, as well as Gene Ontology assignments, are presented in Table S4 and S5, respectively.

## Discussion

Records of mutant phenotypes in *Primula* date back over 400 yr (Gerard, 1597; van de Passe, 1614; Parkinson, 1629) and predominantly affect floral phenotype. More recently identified mutants in *P. sinensis* include flower and leaf phenotypes (De Winton & Haldane, 1933, 1935), some of which are linked to the *S* locus. Contemporary studies in *P. vulgaris* (Webster, 2005) include two phenotypes linked to the *S* locus, *Hose in Hose* (Ernst, 1942; Webster & Grant, 1990; Li *et al*., 2010) and *sepaloid* (Webster, 2005; Li *et al*., 2008); others such as *double* are not linked to the *S* locus (Webster, 2005). *Oakleaf* is the third *S* locus‐linked developmental phenotype in *P. vulgaris*. *Oakleaf* was identified as a spontaneous mutation; it is dominant and affects both flower and leaf morphology. A *P. sinensis* mutation described in 1911, and designated *o* caused oak‐shaped leaves and affected flower morphology, but was recessive and not linked to the *S* locus (Gregory, 1911). The shape and character of the lobed leaves in *Oakleaf* are variable but their presence is characteristic of the mutation. The attenuated petal phenotype is also variable as seen in the F_1_ siblings from an *Oakleaf* × wild‐type cross (Fig. 1d–f). This observation may reflect differences in expressivity of the mutant locus in different organs in response to genetic background.

The mutation sometimes increases separation and size of sepals, but does not cause lobed sepals. Crosses of *Oakleaf* to other floral mutants reveal the organ‐specificity of *Oakleaf* action (Fig. 1). In combination with *Hose in Hose,* both first and second whorls of petals show attenuation characteristic of *Oakleaf* petals (Fig. 1j). In combination with *Jack in the Green*, the leaves that replace sepals have the lobed appearance of *Oakleaf* leaves (Fig. 1k). These two examples, and that of *Oakleaf* combined with *Jackanapes* (Fig. 1l), reveal that *Oakleaf* action is organ‐ and not whorl‐specific. *Oakleaf* does not always affect cotyledons but is consistently presented in the primary leaves. The developmental profile of *Oakleaf* suggests either organ‐specific expression of the dominant locus, or restricted expression, or action, of downstream network components.

Genetic analyses with *Oakleaf* as the female parent, where *Oakleaf* is either in repulsion (Fig. 3a) or coupling (Fig. 4b) to the *S* locus, pollinated from a wild‐type pin, show that *Oakleaf* is inherited as a single dominant locus (Figs 1, 2, 3). However, in the reciprocal crosses, with *Oakleaf* as the male parent (Figs 3b, 4b) we observed progeny numbers that deviated from the anticipated 1 : 1 ratio. In both cases, the missing progeny were consistent with reduced transmission of the dominant thrum *S* allele. Such distorted segregation ratios were not observed in all crosses (Fig. 5): we are unaware of other examples where the pin : thrum ratio distorts from the anticipated equal transmission of dominant and recessive *S* alleles (Darwin, 1862; Bateson & Gregory, 1905). It is therefore unlikely that the distorted ratios are due to poor transmission of the dominant *S* allele.

The data presented in Fig. 3(b) show a significant deviation from the anticipated 1 : 1 ratio (*P *<* *0.001) of *Oakleaf* to wild‐type progeny. The reason for this is unclear, but in this cross 45 seedlings were lost before secondary leaf development. Intriguingly, chi‐squared analysis of progeny numbers, including the 45 lost seedlings as wild‐type, support a 1 : 1 ratio (*P *>* *0.30). *Primula* seedlings are susceptible to ‘damping off’ due to bacterial or fungal infection before secondary leaves emerge. Leaves of *Oakleaf* plants are thicker and firmer than wild‐type. In three of four crosses (Figs 3, 4), progeny losses before flowering were higher for wild‐type than *Oakleaf*. We speculate that if the *Oakleaf* mutation gives greater resilience to seedling loss under unfavourable conditions, or in response to pathogen exposure, this could account for the ratio distortion. Indeed, previous studies of *asymmetric leaves 1* (*as1*) mutants in *Arabidopsis*,* Antirrhinum* and tobacco showed enhanced resistance to necrotrophic fungi (Nurmberg *et al*., 2007). AS1 is involved in repression of *KNOX* gene expression, and *as1* mutants have similar phenotypes to *KNAT1* overexpression lines (Hay *et al*., 2002). This hypothesis for seedling resilience in *Oakleaf* needs to be tested. The reason for underrepresentation of *Oakleaf* progeny in Cross 4 (Fig. 4b) is unclear, and could reflect a statistical consequence of the small progeny numbers.

Based on data obtained from backcrosses (Figs 3a, 4a), and the reciprocal crosses between heterozygous *Oakleaf* plants (Fig. 5) which produce the predicted 1 : 1 and 3 : 1 progeny ratios, respectively, we conclude that *Oakleaf* is caused by a single dominant locus. Linkage of *Oakleaf* to the *S* locus is demonstrated by predominant cosegregation of *Oakleaf* with pin or thrum phenotypes in specific crosses, together with small numbers of recombinants. These crosses suggest a range of potential map distances, but the cross with the largest number of progeny (Fig. 3b) gives a map distance of 3.3 cM. This map distance is possibly an underestimate as the total progeny numbers do not include the 92 plants lost as seedling or before flowering.

By analogy to *Hose in Hose,* where upregulated expression of a transcription factor is responsible for the phenotype (Li *et al*., 2010), and based on similarities to the phenotype of Class I *KNOX* homeodomain gene overexpression in *A. thaliana* (Lincoln *et al*., 1994; Chuck *et al*., 1996; Hay & Tsiantis, 2010), we considered three possibilities as the basis for *Oakleaf* : dominant upregulation of a *PvKNOX* homeodomain gene; mutation in a *PvKNOX* gene that confers a dominant gain of function on the encoded protein, such as a point mutation that introduces an amino acid change, or through a splice site mutation that yields a truncated protein with dominant function; and dominant mutation of a gene unrelated to the *PvKNOX* homeodomain gene family.

We used a combination of *de novo* genome assembly and RNA‐Seq to identify the full complement of eight *PvKNOX* genes (Figs 6b, S1, S2). A fully assembled and annotated *P. vulgaris* genome will form the basis of a future publication. Phylogenetic analysis (Fig. 6b) shows that *P. vulgaris* has five Class I and three Class II *PvKNOX* genes (Kerstetter *et al*., 1994; Bharathan *et al*., 1999). Alignment of RNA‐Seq datasets from *Oakleaf* and wild‐type leaves and flowers enabled us to investigate expression of each gene in *Oakleaf* and wild‐type leaves and flowers (Fig. 7; Table S2). We also explored whether any of the *PvKNOX* genes showed constitutive upregulation in mature leaves and flowers of *Oakleaf*. In line with previous observations on the localised expression of Class I *KNOX* genes in *A. thaliana* (Bharathan *et al*., 1999; Hay & Tsiantis, 2010), we observed low expression of Class I *PvKNOX* genes in wild‐type *Primula* leaves (Fig. 7; Table S2); none is strongly upregulated in *Oakleaf* leaves. Only *PvKNL6* has higher sequence read counts in both *Oakleaf* leaves and flowers (Table S2) but expression in leaves was low with only 15 and 7 reads in *Oakleaf* and wild‐type, respectively. None of the Class I *PvKNOX* genes show strong upregulation in both flowers and leaves of *Oakleaf*.

Analysis of Class II *PvKNOX* gene expression (Fig. 7; Table S2) shows comparable expression levels in leaf and flower tissue and this is consistent with observations of broader expression profiles for Class II *PvKNOX* genes compared with Class I genes (Serikawa *et al*., 1997; Bharathan *et al*., 1999; Truernit *et al*., 2006). In *A. thaliana*, Class II *KNOX* genes have distinct functions from the Class I genes; *KNAT3*,* KNAT4* and *KNAT5* are implicated in root development (Truernit *et al*., 2006) and KNAT7 in secondary cell wall formation (Li *et al*., 2011, 2012). Of the three Class II *PvKNOX* genes, only *PvKNL3* is potentially upregulated in both leaves and flowers of *Oakleaf*; however, because *A. thaliana* Class II *KNOX* genes do not have roles in apical meristem identity, we do not consider *PvKNL3* as a strong candidate for *Oakleaf*. None of the *PvKNOX* genes is strongly upregulated in *Oakleaf* leaves and we conclude that dominant constitutive overexpression of a *PvKNOX* gene is not a basis of the *Oakleaf* phenotype.

In order to establish whether mutation within a *PvKNOX* gene is responsible for *Oakleaf*, we analysed RNA‐Seq read profiles against *PvKNOX* gene models. We speculated that a change in amino acid sequence or expression of a truncated polypeptide might lead to a dominant gain‐of‐function. Analysis of *Oakleaf* RNA‐Seq read profiles for the eight *PvKNOX* genes did not reveal differential splicing between *Oakleaf* and wild‐type that might cause expression of a variant protein. However, several SNPs were identified between *Oakleaf* and the corresponding wild‐type genome sequence. Those SNPs that were homozygous in *Oakleaf*, that were also found in RNA‐Seq data from wild‐type pin flowers, or that were predicted to lead to conservative amino acid substitutions, were discounted as the possible basis for *Oakleaf* (Table S3). Three SNPs in *PvKNL2* and *PvSTL1*, all heterozygous in *Oakleaf,* would cause truncation of the encoded polypeptide, and two further heterozygous SNPs in *PvKNL3* and *PvKNL7* cause nonconservative amino acid substitutions. Although these five SNPs might affect KNOX protein function, those in *PvKNL2* and *PvSTL1* were observed only in *Oakleaf* flower but not leaf transcripts, and those in *PvKNL3* and *PvKNL7* were only observed in *Oakleaf* leaf but not flower transcripts; for *PvKNL7* there were no RNA‐Seq reads over this SNP in flower. It seems unlikely given the absence of the SNP in both flower and leaf samples that these are responsible for the dominant *Oakleaf* phenotype. However, the availability of a *P. vulgaris* genome sequence, and availability of SNPs for each gene will enable future segregation analyses to determine whether any of the *PvKNOX* genes are linked to the *S* locus.

Transcriptome analysis of *Oakleaf* and wild‐type identified cohorts of genes that are differentially up‐ and downregulated. These studies provide not only candidates for genes controlled by *Oakleaf*, but also potential candidates for *Oakleaf* if it proves not to be a *PvKNOX* gene. The 507 genes which are upregulated and 314 genes downregulated (Log_2_ fold cut‐off > 2) (Tables S4, S5) represent a broad spectrum of predicted function and we can only speculate which genes are the likely players in the regulatory networks operating downstream of *Oakleaf*. It has been shown that networks operating downstream of Class I *KNOX* genes in *A. thaliana* involve upregulation of GA2 oxidase and downregulation of GA20 oxidase, alongside upregulation of IPT7, which alter gibberellin and cytokinin concentrations, respectively; genes involved in lignin synthesis such as *COMT1, CCoAOMT* and *AtP12* are also downregulated by Class I *KNOX* genes (Hay & Tsiantis, 2010). Analysis of the differentially expressed genes in *Oakleaf* (Tables S4, S5) does not reveal the *P. vulgaris* homologues for these *A. thaliana* genes. It is possible that *Oakleaf* is not caused by overexpression of a Class I *PvKNOX* gene, but is instead a phenocopy caused by a different pathway, as in the case of *Wavy auricle in blade 1*, a dominant mutant phenotype (Hay & Hake, 2004).

Here we have identified *Oakleaf* as a new *S* locus‐linked phenotype that has enabled us to develop a genetic map of the *S* locus (Li *et al*., 2015). We have explored three possible explanations for the *Oakleaf* phenotype based on analysis of the complete *PvKNOX* gene family and have identified other potential candidates for *Oakleaf*, as well as candidate *Oakleaf*‐regulated genes using RNA‐Seq analysis. Future studies, facilitated by a *Primula* genome assembly and SNP analysis of candidate genes, will reveal potential candidates for *Oakleaf* on the basis of their linkage to the *S* locus.

## Supporting information

Please note: Wiley Blackwell are not responsible for the content or functionality of any supporting information supplied by the authors. Any queries (other than missing material) should be directed to the *New Phytologist* Central Office.

**Fig. S1 **Predicted amino acid sequences of PvKNOX proteins.
**Fig. S2 **Multiple sequence alignment of PvKNOX proteins.
**Table S1 **RNA‐Seq read data from six paired‐end read libraries
**Table S2 **Differential expression of *PvKNOX* genes
**Table S3 **Analysis of single nucleotide polymorphisms in *PvKNL* genes

**Table S4 **Genes upregulated in *Primula vulgaris Oakleaf* as compared with wild‐type
**Table S5 **Genes downregulated in *Primula vulgaris Oakleaf* as compared with wild‐type
