# Supplementary material for: Oakleaf: an S locus‐linked mutation of Primula vulgaris that affects leaf and flower development
Source: New Phytol. 2015 Apr 9;208(1):149–61. doi: 10.1111/nph.13370 (PMC4973830; doi:10.1111/nph.13370)
Supplement: Supplementary file 1 — Fig. S1 Predicted amino acid sequences of PvKNOX proteins. Fig. S2 Multiple sequence alignment of PvKNOX proteins. Table S1 RNA‐Seq read data from six paired‐end read libraries Table S2 Differential expression of PvKNOX genes Table S3 Analysis of single nucleotide polymorphisms in PvKNL genes [file NPH-208-149-s001.pdf]

New Phytologist **Supporting Information Figs S1 & S2 and Tables S1–S3**

Article title: *Oakleaf*: an *S* locus-linked mutation of *Primula vulgaris* that affects leaf and flower development

Authors: Jonathan M. Cocker, Margaret A. Webster, Jinhong Li, Jon Wright, Gemy Kaithakottil, David Swarbreck and Philip M. Gilmartin

Article acceptance date: 07 February 2015

The following Supporting Information is available for this article:

Fig. S1 Predicted amino acid sequences of PvKNOX proteins.

Fig. S2 Multiple sequence alignment of PvKNOX proteins.

Table S1 RNA Seq read data from six paired-end read libraries

Table S2 Differential gene expression of *PvKNOX* genes

Table S3 Analysis of single nucleotide polymorphisms in *PvKNL* genes

Table S4 Genes upregulated in *Primula vulgaris Oakleaf* as compared with wild type (separate Excel file)

Table S5 Genes downregulated in *Primula vulgaris Oakleaf* as compared with wild type (separate Excel file)

AtKNAT1
MEEYQHDNSTTPQRVSFLYSPISSSNKNDNTSDTNNNNNNNNNSSNYGPGYNNTNNNNHHHQHMLFPHMSSLLPQTTENCFRSDHD
QPNNNNNPSVKSEASSSRINHYSMLMRAIHNTQEANNNNNDNVSDVEAMKAKIIAHPHYSTLLQAYLDCQKIGAPPDVVDRITAA
RQDFEARQQRSTPSVSASSRDPELDQFMEAYCDMLVKYREELTRPIQEAMEFIRRIESQLSMLCQSPIHILNNPDGKSDNMGSSD
EEQENNSGGETELPEIDPRAEDRELKNHLLKKYSGYLSSLKQELSKKKKKGKLPKEARQKLLTWWELHYKWPYPSESEKVALAES
TGLDQKQINNWFINQRKRHWKPSEDMQFMVMDGLQHPHHAALYMDGHYMGDGPYRLGP

PvKNL1
MEDYNENTSQSGNFLYGGSLLATTNNNTTSTNTTSSLYGRNSSHQSHMMNMNMNMNMSMNINSFHQLGEYPIVKSEAGGSGTSQN
FIFPNSVIRGIQTGLSNESCSNNDVESIKAKIISHPQYSNLLEAYMDCQKVGAPPEVAAQLTAVREEFEGRQRASTIGKNATKDP
ELDQFMEAYYNMLVKYREELTRPIQEAMDFMRRVEAQLNILNNNNNSTAPVRIFNSEEKCEGVVSSEEDQENSGGETELAEIDPR
AEDQELKNYLLRKYSGYLGSLKQELSKKKKKGKLPKDARQKLLSWWELHYKWPYPSESEKVALAESTGLDQKQINNWFINQRKRH
WKPSEDMQFMVMDGIHPQSASLYMDGHYMGEGPYRLGP

PvKNL2
MDDIYRQVYMSPDNLMMPVDYQALYSSSSASVASGGDHQSRFPFYGSDELISISDQALIRRSTNIHEDDDVLKRKIASHPWYPKL
LDAYIGCQKVGAPPEISCLLEEIQRENDVLNHKAASSCLGEDPELDEFMNTFCEILLKYKSDLSKPLEEATTFLNKIELQFGNLC
KDEGPVSSDDDLGGGEVEGLDNRTQSEEKDLKDRLLRKFGSHISNLKLEFSKKKKKGKLPKEGRQTLLEWWNLHYKWPYPTEGDK
ISLAETTGLDQKQINNWFINQRKRHWKPSENMQLAVMDNLTGQFYAEDD

PvKNL6
MDELYRLHSSISSSDDLTINRLNGNIQFDTIIVREMSDLMKAQIASHPLYPNLVSAYIECRKVGAPPEMALLLDEISKENIPIST
CGEIGVDPELDDFMGTYCAVLQRYKEDLSKPFNEATTFLTSIESQLHDLCKDTIITPTTTSLASDDMAGSSEEEMSCGEVEAVES
QESSVPRKGDKELKEMLLRKYSGYLSSLRKDFLKKRKKGKLPNDARTALLDWWNTHYRWPYPTEEEKVKLSEVTGLDQKQINNWF
INQRKRHWKPSEDMRFALMEGVSGNSNTGSSMYVDHGDGTGSNLVGKQLPRFI

PvSTL1
MSLMNFGENLNSGGANEMMILPNSSSSNNNNTLFQQNHNNFNTSNQNNITPFLLENYNNNQLNVYSTDHQGASSLRAKIIAHPHY
HRLLAAYVNCQKIGAPPEVVAGLEEAYRSVAVRGRVDSSRVGEDPGLDQFMEAYCEMLTKYEQELSKPFKEAMLFLSKIESQFKA
LALPSSDSGFDGYGSSEEEAEFIHNTVDPQAEENELKGQLLRKYSGYLGSLKQEFMKKRKKGKLPKEARNQLLEWWSRNYKWPYP
SESQKMALAESTGLDSKQINNWFINQRKRHWKPSEDMQFVVMDSNSHPHNSAAAHYYMDHVLANPFPMDTSPSFI

PvSTL2
MYANNNSSCSMAFGEHLNSGGIYPMMTMPNSSPSNNNTLFENNHTSENQTSFFMENNQNINSNDDHGGSTSASSISLRAKIMAHP
HYHRLLAAYVNCQKIGAPQEVVARLEEAYQVMIGRVGPGCVGDDPGLDQFMEAYCEMLNKYEQELSKPFKEAMLFLSKFESQFRA
LTLSSTGHSGFERNGSSEEEADTDYSIVDRQEEDKDLKGRLLRKYSGYLGSLKQEFMKKKKKGKLPKEARENLLEWWSRNHKWPY
PSESQKMELAESTGLDPKQINNWFINQRKRHWKPTDEMQFVMMDPNSNPNAAAHHYYMDHVMVNPFPMDI

PvKNL3
MDMQEMAGLHQHYSDQQLSDTSSVLRSITVDGSTDDQQNPHHQQQQGGPTWLNSAILQTNFLNLQTNSESPTSRPHHHQGHNNSN
AGQWTPRSMDTNNNNNNNNNNNNNNNNNNNNNNESVVEDPGGSMSWQTARHKAEILAHPLYEQLLSAHVACLRIATPVDQLPRIDAQL
AQSQQVVAKYSGMAHGGISMGDDKELDQFMTHYVLLLCSFKEQLQQHVRVHAMEAVMACWELEQSLQSLTGVSPGEGTGATMSDD
EEDQLESDNNLFDGSLDGHDSTGFGPLVPTESERSLMERVRQELKHELKQGYKEKIVDIREEILRKRRAGKLPGDTTSVLKAWWQ
SHSKWPYPTEEDKARLVQEPGLELKQINNWFINQRKRNWHSNPSTSTILKSKRKSSSGDKNRDQFA

PvKNL4
MAFHHTQELPLHHFSDDSSGGPPPTWLNSEILRQNHYGGTGGRSFLQTINNNNNNNNSENNDEFSRRSESDGSGEEREWQTAKCK
SDILSHPLYEQLLSAHVQCLRIATPVDQLPRIDAQLAQSQQVVAKYSVLGHGNPPLDDKDLDQFMTHYVLLLSSFKEQLQQHVRV
HAMEAVMACWELEQSLQSLTGVAPGEGTGATMSDDDEDQADSDTNIYDGSLDGTDNMGFGMPTESERSLMARVRQELKLELKQGY
KEKIVDIREEILRKRRAGKLPGDTTSHLKAWWQSHSKWPYPTEEDKARLVQETGLQLKQINNWFINQRKRNWHSNPSSSTVVKSK
RKSNAGEPSNERFM

PvKNL7
MQEQGGNMGMMGGGDHFSGDVGGGEMKAEIAAHPLCEQLLAAHVACLRVATPIDQLPMIEAQLAQAQGIVRAYAASVQGNRPLSP
HERQELDNFLGQYIMVLSSFKDQLQQHVRVHAVEAVMACREIEHNLQALTGVTLGEGSGATMSDDEADDTNNIMQQMDFSMDHQA
SAEGHDMMGFGPLLPTESERTLMERVRQELKIELKQGFRSRIEDVREEILRKRRAGKLPGDTTSVLKNWWQLHAKWPYPTEDDKA
KLVEETGLQLKQINNWFINQRKRNWHNNSQSVTALKSKRKR

Fig. S1 Predicted amino acid sequences of PvKNOX proteins**.** Amino acid sequences of wild type PvKNOX proteins are shown together with the *Arabidopsis thaliana* KNAT1 (AtKNAT1) protein sequence. Names of *Primula* proteins reflect similarity to *A. thaliana* KNAT and STM proteins. The standard single letter amino acid code is used. The sequences were used to generate the phylogenetic tree (Fig. 6b) and protein sequence alignments (Fig. S2).

```
AtKNAT1 ---------MEEYQHDN---------------S---------------TTPQRVSFLYSP   21
PvKNL1  ---------MEDYNEN---------------------------------TSQSGNFLYGG   18
PvKNL2  ------------------------------------------------------------   0
PvKNL6  ------------------------------------------------------------   0
PvSTL1  ------------------------------------------------------------   0
PvSTL2  ------------------------------------------------------------   0
PvKNL3  MDMQEMAGLHQHYSDQQLSDTSSVLRSITVDGSTDDQQNPH-----HQQQQGGPTWLNSA   55
PvKNL4  ---------------------------MAFH---HTQELPLHHFSDDSSGGPPPTWLNSE   30
PvKNL7  ------------------------------------------------------------   0

AtKNAT1 ISSSNKNDNTSDTNNNNNNNNSSNYGPGYNNTNNNNHHHQHMLFPHMSSLLPQTTENCFR   81
PvKNL1  SLLATTNNNTTST-----NTTSSLYGRN-------SSHQSHMMNMNMNMNMSM-NINSFH   65
PvKNL2  ----------------------------------MDDIYRQVYMSPDNLMMPVDYQALYS   26
PvKNL6  ------------------------------------------------------MDELYR   6
PvSTL1  -------------------MSLMNFGENLNSGG----ANEMMILPNSSS--SNNNNTLFQ   35
PvSTL2  ------------MYANNNSSCSMAFGEHLNSGG----IYPMMTMPNSSP--SNN-NTLFE   41
PvKNL3  ILQTNFLNLQT-------NSESPTSRPHHHQGHNN---------SNAGQWTPR----SMD   95
PvKNL4  ILRQNHYGGT------------------------------------GG----R----SFL   46
PvKNL7  ----------------------------------------------------------MQ   2

AtKNAT1 SDHDQPNNNNNPSVKSEASSSRIN-HYSMLMRAIH---NTQEANNNNNDNVSDVEAMKAK   137
PvKNL1  Q------LGEYPIVKSEAGGSGTSQNFIFPNSVIR---GIQTGLSNESCSNNDVESIKAK   116
PvKNL2  SSS-----------ASVASGGDHQSRFPFYGSDELISISDQALIRRSTNIHEDDDVLKRK   75
PvKNL6  L-------------HSSISSSDDLT-INRLNGN------IQF---DTIIVREMSDLMKAQ   43
PvSTL1  QNHNNFN------------TSNQNNITPFLLENYN---NNQLNV--YSTDHQGASSLRAK   78
PvSTL2  NNHTSE------------------NQTSFFMENNQ---NINSNDDHGGSTSASSISLRAK   80
PvKNL3  TNNNNNNNNNN---------NNNNNNNN----------NESVVEDPGGSMSWQTARHKAE   136
PvKNL4  QTINNNNNNNN---------SENNDEFS----------RRSESDGSGEEREWQTAKCKSD   87
PvKNL7  ---------------------EQGGNM------------GMMGGGDHFSGDVGGGEMKAE   29

                        Helix Loop Helix
AtKNAT1 IIAHPHYSTLLQAYLDCQKIGAPPDVVDRITAARQDFEARQQR-STP----SVSASSRDP   192
PvKNL1  IISHPQYSNLLEAYMDCQKVGAPPEVAAQLTAVREEFEGRQRA-STI----G-KNATKDP   170
PvKNL2  IASHPWYPKLLDAYIGCQKVGAPPEISCLLEEIQRENDVLNH---KA----ASSCLGEDP   128
PvKNL6  IASHPLYPNLVSAYIECRKVGAPPEMALLLDEISKENIPIS----------TCGEIGVDP   93
PvSTL1  IIAHPHYHRLLAAYVNCQKIGAPPEVVAGLEEAYRSVAVRG----RV----DSSRVGEDP   130
PvSTL2  IMAHPHYHRLLAAYVNCQKIGAPQEVVARLEEAYQVM--IG----RV----GPGCVGDDP   130
PvKNL3  ILAHPLYEQLLSAHVACLRIATPVDQLPRIDAQLAQSQQVVAKYSGMAHGGISMG--DDK   194
PvKNL4  ILSHPLYEQLLSAHVQCLRIATPVDQLPRIDAQLAQSQQVVAKYSVLGHGNPPL---DDK   144
PvKNL7  IAAHPLCEQLLAAHVACLRVATPIDQLPMIEAQLAQAQGIVRAYAASVQGNRPLSPHERQ   89
        * :**    *: *:: * ::.:* :    :

                      Amphipathic Helix H3
AtKNAT  ELDQFMEAYCDMLVKYREELTRPIQ----EAMEFIRRIESQLSMLCQ-----SPIHILNN   243
PvKNL1  ELDQFMEAYYNMLVKYREELTRPIQ----EAMDFMRRVEAQLNILNNNNNSTAPVRIFNS   226
PvKNL2  ELDEFMNTFCEILLKYKSDLSKPLE----EATTFLNKIELQFGNLCKDEGPVSS------   178
PvKNL6  ELDDFMGTYCAVLQRYKEDLTKPFN----EATTFLTSIESQLHDLCKDTIITPTTTSLAS   149
PvSTL1  GLDQFMEAYCEMLTKYEQELSKPFK----EAMLFLSKIESQFKALALPS-SDS-------   178
PvSTL2  GLDQFMEAYCEMLNKYEQELSKPFK----EAMLFLSKFESQFRALTLSSTGHS-------   179
PvKNL3  ELDQFMTHYVLLLCSFKEQLQQHVRVHAMEAVMACWELEQSLQSLTGVSPGEGTGATMSD   254
PvKNL4  DLDQFMTHYVLLLSSFKEQLQQHVRVHAMEAVMACWELEQSLQSLTGVAPGEGTGATMSD   204
PvKNL7  ELDNFLGQYIMVLSSFKDQLQQHVRVHAVEAVMACREIEHNLQALTGVTLGEGSGATMSD   149
         **:*:  :  :*  :..:* : ..    **      .* .:  *
                                                            ELK Domain
AtKNAT1 PDGK--------S---DNMGSSDEEQENNSGGETELP----EIDPRAEDRELKNHLLKKY   288
PvKNL1  EE-K--------C---EGVV-SSEEDQENSGGETELA----EIDPRAEDQELKNYLLRKY   269
PvKNL2  -------------------------DDDLGGGEVEGL----DNRTQSEEKDLKDRLLRKF   209
PvKNL6  ----------------DDMAGSSEE--EMSCGEVEAVESQESSVPRKGDKELKEMLLRKY   191
PvSTL1  ---G--------F---DGYGSSEEE------AEFIHN----TVDPQAEENELKGQLLRKY   214
PvSTL2  ---G--------F---ERNGSSEEE------ADTDYS----IVDRQEEDKDLKGRLLRKY   215
PvKNL3  DEEDQLESDNNLF------DGSLDGHDSTGFGPLVPTES-ERSLMERVRQELKHELKQGY   307
PvKNL4  DDEDQADSDTNIY------DGSLDGTDNMGFG--MPTES-ERSLMARVRQELKLELKQGY   255
PvKNL7  DEADDTNNIMQQMDFSMDHQASAEGHDMMGFGPLLPTES-ERTLMERVRQELKIELKQGF   208
                                                         .:**  * : :

           ELK Domain                           Homeodomain
AtKNAT1 SGYLSSLKQELSKKKKKGKLPKEARQKLLTWWELHYKWPYPSESEKVALAESTGLDQKQI   348
PvKNL1  SGYLGSLKQELSKKKKKGKLPKDARQKLLSWWELHYKWPYPSESEKVALAESTGLDQKQI   329
PvKNL2  GSHISNLKLEFSKKKKKGKLPKEGRQTLLEWWNLHYKWPYPTEGDKISLAETTGLDQKQI   269
PvKNL6  SGYLSSLRKDFLKKRKKGKLPNDARTALLDWWNTHYRWPYPTEEEKVKLSEVTGLDQKQI   251
PvSTL1  SGYLGSLKQEFMKKRKKGKLPKEARNQLLEWWSRNYKWPYPSESQKMALAESTGLDSKQI   274
PvSTL2  SGYLGSLKQEFMKKKKKGKLPKEARENLLEWWSRNHKWPYPSESQKMELAESTGLDPKQI   275
PvKNL3  KEKIVDIREEILRKRRAGKLPGDTTSVLKAWWQSHSKWPYPTEEDKARLVQEPGLELKQI   367
PvKNL4  KEKIVDIREEILRKRRAGKLPGDTTSHLKAWWQSHSKWPYPTEEDKARLVQETGLQLKQI   315
PvKNL7  RSRIEDVREEILRKRRAGKLPGDTTSVLKNWWQLHAKWPYPTEDDKAKLVEETGLQLKQI   268
           : .:: :: :*:: **** :    *  **. . :****:* :*  * :  **: ***

            Homeodomain
AtKNAT1 NNWFINQRKRHWKPSEDMQFMVMDGLQHPHHAA---LYMDGHYMGDGPYRLGP-------   398
PvKNL1  NNWFINQRKRHWKPSEDMQFMVMDGI-HPQSAS---LYMDGHYMGEGPYRLGP-------   378
PvKNL2  NNWFINQRKRHWKPSENMQLAVMDNLTGQFYAEDD-------------------------   304
PvKNL6  NNWFINQRKRHWKPSEDMRFALMEGVSGNSNTGSS-MYVDHGD-GTGSNLVGKQLPRFI    308
PvSTL1  NNWFINQRKRHWKPSEDMQFVVMDSNSHPHNSAAAHYYMDHVLANPFPMDTSPSFI----   330
PvSTL2  NNWFINQRKRHWKPTDEMQFVMMDPNSNPN-AAAHHYYMDHVMVNPFPMDI---------   325
PvKNL3  NNWFINQRKRNWHSNPSTSTILKSKRKSSSGDKNRDQFA---------------------   406
PvKNL4  NNWFINQRKRNWHSNPSSSTVVKSKRKSNAGEPSNERFM---------------------   354
PvKNL7  NNWFINQRKRNWHNNSQSVTALKSKRKR--------------------------------   296
        **********.*: .
```

Fig. S2 Multiple sequence alignment of PvKNOX proteins. Clustal Omega multiple sequence alignment of the eight predicted wild type PvKNOX proteins, and *Arabidopsis thaliana* KNAT1 (AtKNAT1) is shown. The standard single letter amino acid code is usedAmino acid sequence numbers for each line are indicated at right. The final number in brackets indicates the full length amino acid sequence of each protein. The Helix Loop Helix domain is indicted in red, the Amphipathic Helix H3 in blue, the ELK domain in green, and the Homeodomain in orange. (-), alignment gaps. (*), fully conserved residues; (:), strongly similar property residues; (.),weakly similar property residues.

Table S1 RNA-Seq read data from six paired-end read libraries

Sample	Paired-end reads	Total no. of bases	Transcript assemblies
Oakleaf open flowers	24,995,179	5.0×10^{9}	33,197
wild type open flowers	33,400,153	6.7×10^{9}	35,809
Oakleaf young leaves	14,310,589	2.9×10^{9}	27,457
wild type young leaves	45,723,021	9.1×10^{9}	33,711
Mixed stage pin flowers	100,768,798	2.0×10^{10}	39,688
Mixed stage thrum flowers	81,045,610	1.6×10^{10}	41,488

The number of paired-end sequence reads, the total number of bases and the number of transcript assemblies obtained from each of the six tissue samples are shown, sequences <200 bp have not been included in the transcript assembly numbers.

Table S2 Differential gene expression of *PvKNOX* genes

Primula vulgaris gene	*Arabidopsis thaliana gene*	*Knox gene Class*	Normalised read counts				Log$_2$ fold change	
			Leaf		Flower			
			Oakleaf	wild type	*Oakleaf*	wild type	leaf	flower
PvKNL1	*KNAT1*	I	0	1	307	194	–	0.66
PvKNL2	*KNAT2*	I	27	73	232	135	-1.45	0.78
PvKNL6	*KNAT6*	I	15	7	1502	1077	0.96	0.48
PvSTL1	*STM1*	I	2	0	135	79	–	0.77
PvSTL2	*STM1*	I	0	0	558	255	–	1.13
PvKNL3	*KNAT3*	II	1971	1591	1704	1424	0.31	0.26
PvKNL4	*KNAT4*	II	1716	3368	1232	2595	-0.97	-1.07
PvKNL7	*KNAT5*	II	2690	672	1690	3056	2.00	-0.85

The eight *P. vulgaris KNOX* genes, identified from gene models based on assembled *P. vulgaris* genome sequence data and RNA-Seq transcript assemblies, were named according to the similarity of their encoded proteins to *Arabidopsis* KNOX proteins. *Primula vulgaris* genes are shown alongside the corresponding *A. thaliana* gene and its unigene identifier (ID). RNA-Seq normalized read counts from leaf and flower tissues of *Oakleaf* and wild type plants are shown; the log$_2$ fold change in expression between the leaf samples and flower samples is shown. The wild type was a pin plant.

Table S3 Analysis of single nucleotide polymorphisms in *PvKNL* genes

Gene	Position in genomic DNA	Base Change	Amino acid change	Reads for *Oak* SNP in flower	Total reads at this position	Reads for *Oak* SNP in leaf	Total reads at this position	Genotype and Impact of SNP on encoded protein
PvKNL1	671	TCT - ACT	Ser35 - Thr	7	7	0	0	Homozygous
PvKNL1	1882	ACG - ATG	Thr161 - Met	3	3	0	0	Homozygous
PvKNL2	3534	GCT - GGT	Ala120 - Gly	3	3	No SNP	No SNP	Homozygous
PvKNL2	25367	GGG - GCG	Gly233 - Ala	9	10	No SNP	No SNP	Also found in pin
PvKNL2	30146	CAG - TAG	Gln298 - Stop	2	15	No SNP	No SNP	Truncated protein
PvSTL1	8202	Frameshift -2 bp	aa12: stop16	3	8	No SNP	No SNP	Truncated protein
PvSTL1	8188	GAA - GCA	Glu17 - Ala	8	15	2	2	Substitution tolerated
PvSTL1	8068	Frameshift +2 bp	aa57: stop63	4	11	No SNP	No SNP	Truncated protein
PvSTL1	2413	TCG - CCG	Ser271 - Pro	5	10	No SNP	No SNP	Substitution tolerated
PvSTL1	2278	GTC - ATC	Val316 - Ile	8	13	No SNP	No SNP	Substitution tolerated
PvSTL2	7432	GAA - CAA	Glu47 - Gln	37	37	0	0	Homozygous and in Pin
PvSTL2	7336	GCG - ACG	Ala79 - Thr	29	29	0	0	Homozygous and in Pin
PvSTL2	3225	ATT - ATG	Ile197 - Met	31	31	0	0	Homozygous and in Pin
PvKNL3	7545	CAG - CAT	Gln65 - His	67	98	50	63	Substitution tolerated
PvKNL3	2860	TTG - TCG	Leu335 - Ser	No SNP	No SNP	2	15	Substitution not tolerated
PvKNL3	2034	AGT - AGA	Ser395 - Arg	No SNP	No SNP	2	11	Also found in pin
PvKNL4	2083	AAC - AGC	Asn28 - Ser	17	69	23	66	Substitution tolerated
PvKNL7	8564	GGG - GAG	Gly6 - Glu	0	0	66	132	Substitution not tolerated

Single nucleotide polymorphisms (SNPs) detected in *PvKNL* genes, as compared with the wild type draft genome sequence. The position of each polymorphism in the genomic DNA is indicated. The resulting codon and amino acid (aa) changes are also shown. For frameshifts, the aa at which the peptide sequence diverges from wild type is shown, as is the next stop codon in the shifted reading frame. The Illumina read counts with the *Oakleaf* SNP variant, are shown for flower and leaf transcriptomes. The total number of reads at this position (*Oakleaf* and wild type) are also shown. Where no SNP was detected in either flower or leaf RNA-Seq data this is indicted as ‘No SNP’. The final column indicates whether the *Oakleaf* SNP as compared with the wild type draft genome is homozygous or heterozygous in *Oakleaf*. Where the same *Oakleaf* SNP was also found in the wild type pin plant transcriptome data this is also indicated. Where the SNP is heterozygous, the impact of the SNP on the encoded protein as determined by SIFT (Ng & Henikoff, 2003) (http://sift.bii.a-star.edu.sg/) is shown.

Reference

Ng PC, Henikoff S. 2003. SIFT: predicting amino acid changes that affect protein function. *Nucleic Acids Research* **31:** 2812–3814.
